# Supplementary material for: The Impact of Varieties and Growth Stages on the Production Performance and Nutritional Quality of Forage Triticale in the Qaidam Basin
Source: Plants (Basel). 2025 Sep 23;14(19):2942. doi: 10.3390/plants14192942 (PMC12526206; doi:10.3390/plants14192942)
Supplement: Supplementary file 1 [file plants-14-02942-s001.zip › plants-3865207-supplementary.pdf]

Table S1. The growth stage of different triticale varieties in 2024 and 2025.

Variety	Phenological stages (day)							
	Record of 2024				Record of 2024			
	Booting stage	Heading stage	Flowering stage	Milking stage	Booting stage	Heading stage	Flowering stage	Milking stage
QSM-1	8	8	14	18	9	9	11	18
QSM-2	10	7	13	18	9	8	11	18
QSM-3	9	8	13	22	10	10	11	18
QSM-7	11	6	16	21	10	9	14	19
QSM-8	11	7	14	21	9	9	14	18
JSM-2	10	7	16	21	8	9	14	19
JSM-3	9	8	15	20	11	10	15	18
